# Role of Color Doppler in Scrotal Lesions

**DOI:** 10.4103/0971-3026.54874

**Published:** 2009-08

**Authors:** Bhardwaj Patiala

**Affiliations:** Department of Radiodiagnosis, Govt. Medical College and Rajindra Hospital, Patiala, India

**Keywords:** Color Doppler, testicular torsion, epididymo-orchitis, varicoceles

## Abstract

Color Doppler USG of the scrotum has been demonstrated to be useful in the diagnosis of scrotal lesions. Gray-scale USG characterizes the lesions as testicular or extratesticular and, with color Doppler and power Doppler, flow and perfusion can also be assessed. Color Doppler is particularly helpful in acute painful conditions, where it can differentiate testicular ischemia from inflammatory conditions and thus prevent unnecessary surgical explorations. With color Doppler, useful information can be gained about vascularity in testicular malignancies. Color Doppler also has high sensitivity and high specificity in the diagnosis of lesions like varicoceles.

## Introduction

Scrotal lesions can be broadly classified as testicular and extratesticular. The common testicular lesions are torsion, trauma, neoplasms and inflammatory conditions. Extratesticular lesions include lesions of the spermatic cord, epididymis and scrotal wall. In clinical practice, color Doppler imaging of the scrotum is mainly indicated in acute painful scrotal conditions and assessment of varicoceles.[1] Epididymo-orchitis and testicular torsion have similar clinical presentations and color Doppler is useful in accurately differentiating between the two.[2] Testicular viability can also be very well assessed with color Doppler in cases of testicular trauma.[3]

## Discussion

The common indications of color Doppler USG are in the acute scrotum, for evaluation of varicoceles and for assessing the vascularity of testicular malignancies.

### Imaging of the acute scrotum

The commonly encountered cases in clinical practice are testicular torsion, acute epididymo-orchitis, acute epididymitis, primary orchitis, testicular trauma and torsion of testicular appendages.

### Testicular torsion

**Figure 1 (A, B):**
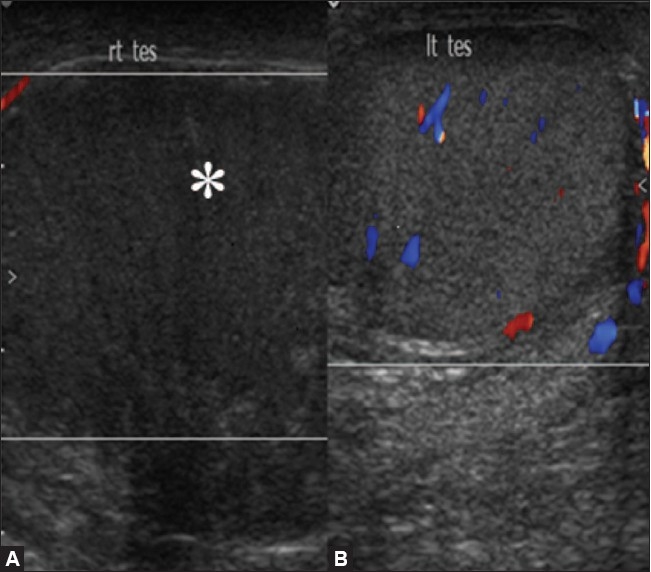
Torsion. Color Doppler shows a hypoechoic and enlarged right testis (arrow in A) showing absence of color flow with normal color flow and echo pattern in the left testis (B)

Gray-scale USG along with color Doppler plays a pivotal role in differentiating testicular torsion from acute epididymo-orchitis and helps in avoiding unnecessary surgery. Both these conditions present with similar clinical features and there is a false positive rate of 50% for the diagnosis of testicular torsion based on clinical findings alone.[4] Testicular torsion occurs due to twisting of the spermatic cord. Two types of testicular torsion are described: intravaginal and extravaginal. Extravaginal torsion occurs exclusively in neonates. Intravaginal torsion occurs within the tunica vaginalis and is due to the presence of a long and narrow mesentery or because of a bell-clapper deformity, which causes the testis to freely swing and rotate within the tunica vaginalis, much like a clapper inside a bell. The bell-clapper deformity is bilateral in most cases.[5] Testicular salvage is possible if treatment is initiated within 4–6 h of torsion.[6] Depending on the extent of twisting (which may range from 180 to 720°) and the duration of the torsion, a wide spectrum of findings may be seen in these cases. The most common findings on gray-scale USG done 4–6 h after the onset of torsion are testicular swelling and decreased echogenicity [Figure 1]. Heterogeneous echotexture is seen 24 h after the onset of torsion and is due to hemorrhage and infarction. In the acute stage, the testis may only show enlargement with a normal echotexture and so color Doppler and power Doppler examination are important to rule out decreased or absent flow[7] [Figure 1]. The spermatic cord immediately cranial to the testis and epididymis is twisted, which gives it a characteristic ‘torsion knot’ or ‘whirlpool appearance’.[8] In torsion of the testicular appendages, color Doppler shows increased peripheral flow around the twisted appendage but testicular perfusion is normal. There are some pitfalls in the diagnosis of torsion with color Doppler; for example, smaller degrees of torsion may be missed on color Doppler; also, in torsion–detorsion, the testis may be hyperemic, which may lead to a false diagnosis of inflammation.[9]

### Epididymitis and epididymo-orchitis

**Figure 2 (A, B):**
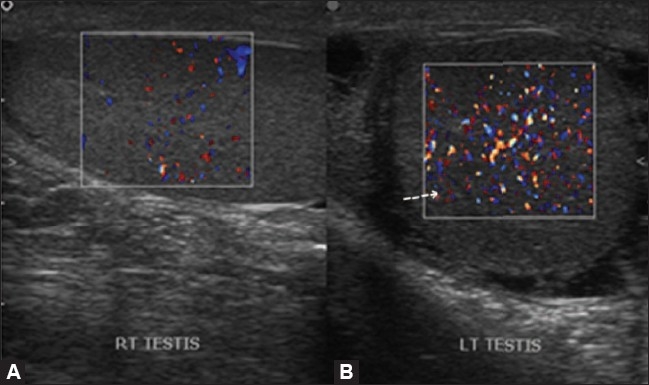
Orchitis. Color Doppler shows increased flow in the left testis (arrow in A) with normal color flow in the right testis

**Figure 3 (A,B):**
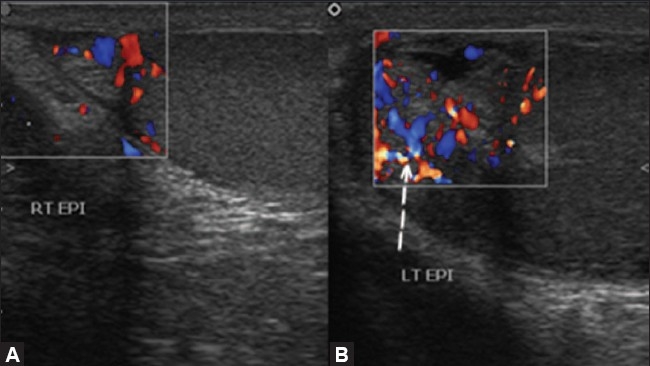
Epididymitis, Color Doppler shows an enlarged left epididymis with increased color flow (arrow in B), suggestive of epididymitis. The right epididymis is normal in size and shows normal color flow (A)

Epididymo-orchitis occurs due to retrograde infection from the bladder or the prostate gland. The epididymal head is the region most commonly affected,[1] appearing enlarged and hypoechoic on gray-scale USG [Figure 2]. However, it may also show normal or increased echogenicity, depending on the duration of the disease. Color Doppler examination shows increased vascularity in the epididymis or in both, the testis and epididymis[10] [Figures 2 and 3]. A reactive hydrocele is usually seen. On spectral Doppler, a low-resistance waveform is seen. Isolated orchitis is very rare and, when present, shows an enlarged hypoechoic testis with increased vascularity on color Doppler examination.[10]

### Testicular trauma

**Figure 4 (A,B):**
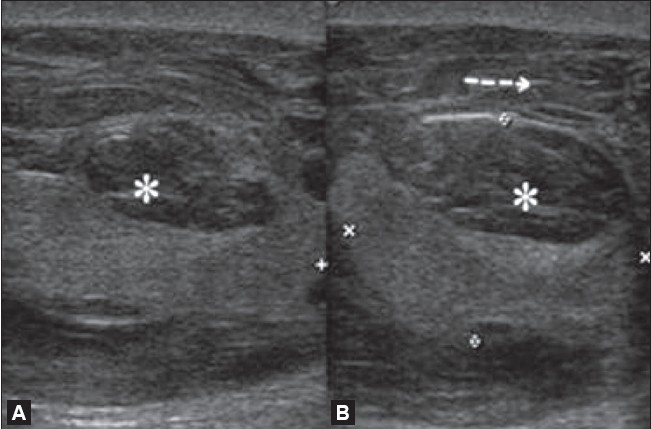
Testicular trauma.USG shows a focal hypoechoic area in the testis (asterix in A) in a case of testicular trauma, suggestive of focal laceration. Thickening of the scrotal wall is also seen due to edema (arrow in B)

**Figure 5:**
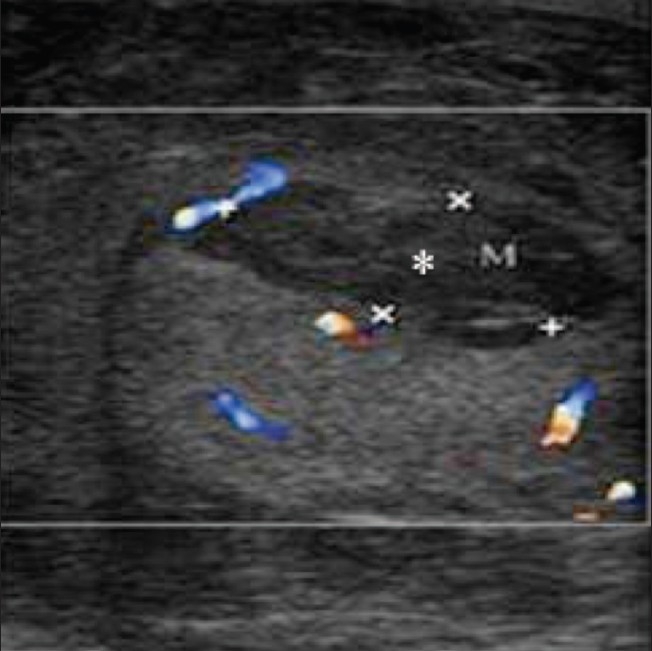
Testicular trauma. Color Doppler shows no color flow in the focal lesion (asterix) due to trauma

Testicular trauma occurs most often after motor vehicle accidents or sports injuries. The findings may include contusion, fracture or rupture of the testis and extratesticular hematoma. Gray-scale examination shows an enlarged testis with a heterogenous echotexture and ill-defined margins [Figure 4]. Hydrocele, hematocele, scrotal wall thickening and rupture of the tunica albuginea are the other findings that may be seen.[11,12] Involvement of capsular vessels, traumatic testicular infarction, etc., can be assessed very well with color Doppler examination. In cases of trauma, areas of testicular infarction or laceration show absence of vascularity on color Doppler imaging. Hematomas appear hypoechoic or as complex collections, with internal echoes and septae; they are avascular on color Doppler USG [Figure 5].[13]

### Varicoceles

**Figure 6 (A,B):**
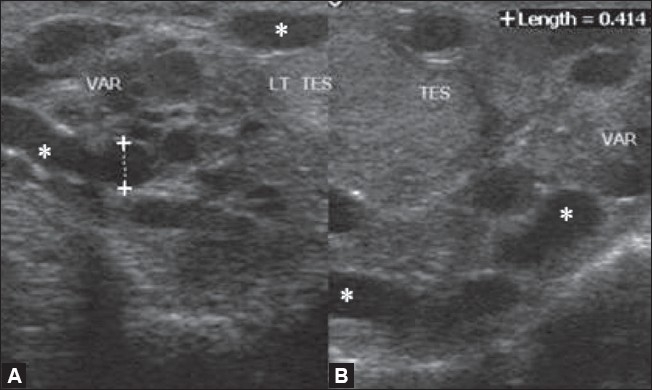
Varicoceles. USG shows multiple dilated tortuous anechoic channels superior and posterior to the testis measuring more than 2 mm in size (asterix in A), suggestive of varicocoeles. Tortous, anechoic channels are also seen within the testis indicating intratesticular varicoceles (B)

**Figure 7:**
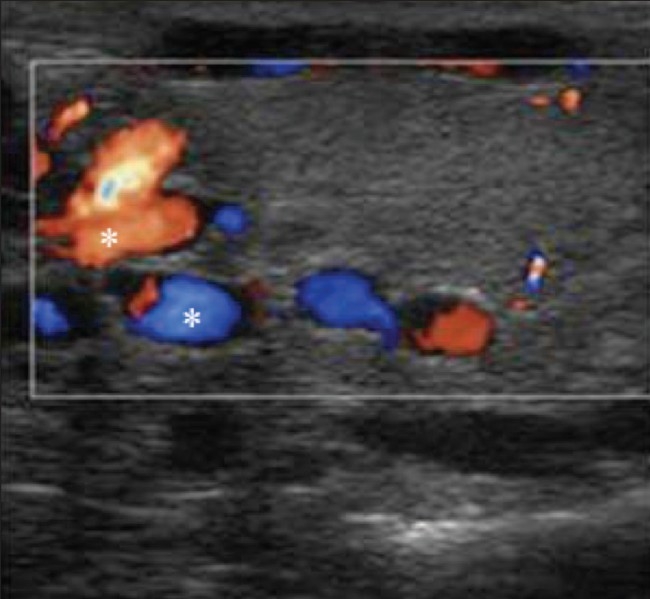
Varicoceles Color Doppler shows a venous pattern of color flow in the anechoic channels (asterix) confirming the diagnosis of both intratesticular and extratesticular varicoceles

Varicoceles are abnormally dilated (more than 2 mm in diameter) and tortuous veins of the pampiniform plexus. They are generally seen posterior and lateral to the testis within the spermatic cord. The normal diameter of the veins of the pampiniform plexus ranges from 0.5 to 1.5 mm. Varicoceles are of two types: primary (or idiopathic) and secondary. Idiopathic or primary varicoceles are caused by incompetence of valves of the internal spermatic vein.[14] They are more common on the left side because of the longer course of the left testicular vein, which joins the left renal vein at a right angle and, in some cases, because of the compression of the left renal vein by the left testicular artery. Increased pressure on the spermatic vein or its tributaries by lesions such as abdominal masses and severe hydronephrosis causes secondary varicoceles.[15] Varicocele is an important cause of infertility, and surgical treatment can bring about a 50% improvement in sperm quality[16] On gray-scale USG, varicoceles appear as multiple serpiginous, tubular (≥2 mm diameter), hypoechoic structures of varying size [Figure 6]. They are generally seen posterior or lateral to the testis. Color Doppler USG reveals the typical venous flow pattern [Figure 7]. Retrograde filling of these varices can be very well demonstrated on color or power Doppler. Spectral Doppler demonstrates slow flow and phasic variation in flow. These are better demonstrated by having the patient performing the Valsalva maneuver. Occasionally, varicoceles may be only intratesticular in location and color Doppler clearly demonstrates the venous flow pattern.[10]

### Testicular tumors

**Figure 8:**
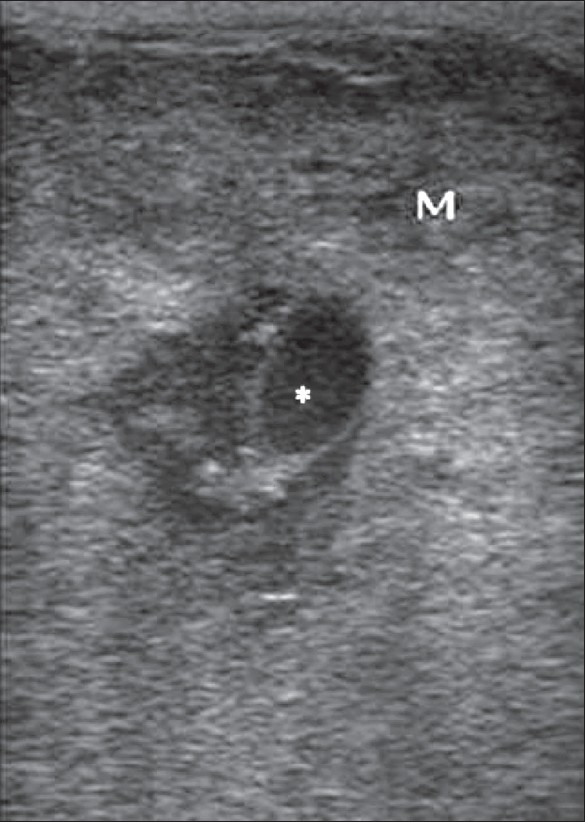
Non-seminomatous germ cell tumor. USG shows a focal lesion having a heterogenous echotexture with solid and cystic components (asterix)

**Figure 9:**
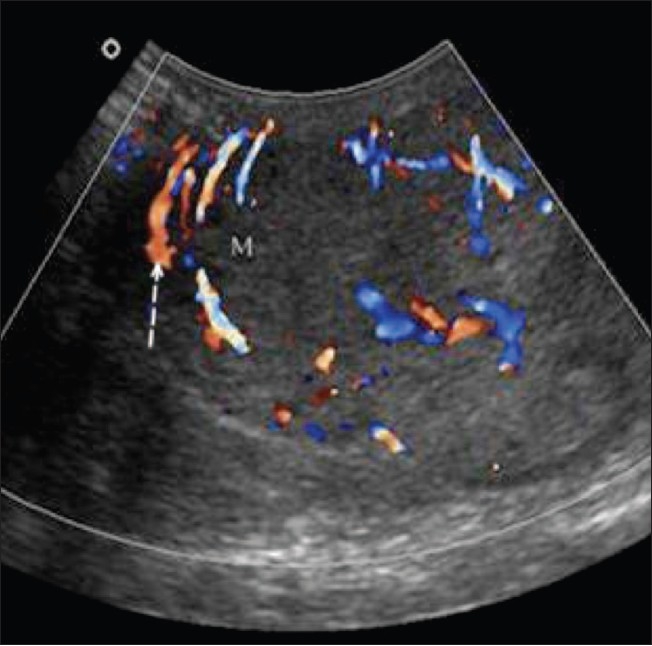
Non-seminomatous germ cell tumors. Color Doppler shows a focal lesion with increased vascularity (arrow)

Most primary testicular malignancies are germ cell tumors and, among the germ cell tumors, seminoma is the most common.[10] The other malignant testicular tumors are stromal tumors, mixed germ cell–stromal tumors, leukemia, lymphoma and metastases.[17] The most common appearance of a seminoma on gray-scale USG is as a homogenous hypoechoic lesion. Most testicular tumors are hypoechoic in echotexture, though they may sometimes also be hyperechoic. Nonseminomatous germ cell tumors show heterogenous attenuation [Figure 8]. Color Doppler assessment provides information about the vascularity of tumor [Figure 9]. Large-sized lesions are hypervascular, whereas smaller ones are hypovascular.[18] Testicular lymphomas show diffuse hypoechogenicity of the testis or present as focal hypoechoic areas. Color Doppler imaging shows increased vascularity.[19]

## Conclusion

Gray-scale USG is an accurate, fast and useful imaging modality for the imaging of scrotal lesions. Gray-scale USG can distinguish between intratesticular or extratesticular lesions and also aid in the characterization of various lesions. Color Doppler enhances the visualization of varicoceles. Color Doppler USG is the modality of choice to differentiate testicular torsion from inflammatory conditions and can thus help in avoiding unnecessary surgical explorations.
